# A Special Design to Facilitate Retrieval of Cement-Retained Zirconia-Based Implant-Supported Restorations

**DOI:** 10.30476/DENTJODS.2020.85080.1114

**Published:** 2021-06

**Authors:** Mitra Farzin, Reza Derafshi, Rashin Giti, Masumeh Taghva

**Affiliations:** 1 Dept. of Prosthodontics, School of Dentistry, Shiraz University of Medical Sciences, Shiraz, Iran; 2 Dept. of Prosthodontics, Biomaterials Research Center, School of Dentistry, Shiraz University of Medical Sciences, Shiraz, Iran

**Keywords:** Zirconia, Implant-Supported, Retrieval

## Abstract

**Statement of the Problem:**

Retrieval of cement-retained implant-supported restorations is challenging in cases of screw loosening or periodontal problems.

**Purpose:**

The purpose of this study was to evaluate the effect of the screw access hole on the fracture resistance of zirconia-based cement-retained restorations with and without an access opening.

**Materials and Method:**

In this *in vitro* study thirty-three cement-retained implant-supported zirconia-based molar crowns were fabricated and divided into 3 groups (n=11). As the control group, group 1 consisted of conventional cement-retained crowns. Group 2 comprised conventional cement-retained crowns in which a hole was created in the location of the screw. Group 3 consisted of cement-retained crowns in which a ledge was created in the location of the screw access channel. The specimens were cemented to their abutments and their access openings were filled with composite resin. A compressive load was applied to the specimens using a universal testing machine until they fractured. The mean fracture resistance values of the samples were compared by using the one-way ANOVA and Tamhane post-hoc test (a=0.05).

**Results:**

The mean fracture resistance values were 1270.18± 12.67 N in group 1 (the control group), 960.09±210.67 N in group 2 (conventional), and 1357.81±361.68 N in group 3 (the special design). The fracture resistance value was higher in the special design group than that of the conventional design (*p*= 0.018) and the fracture resistance value of the conventional design group was less than that of the control group (*p*= 0.042). No statistically significant difference was detected between the control group and the special design group in fracture resistance values.

**Conclusion:**

Preparing a screw access hole in cement-retained implant-supported zirconia-based crowns decreased the fracture resistance of the restoration. Designing a ledge in the zirconia framework around the access hole may increase the fracture resistance of the restoration.

## Introduction

Dental implants are considered to be a scientifically and clinically proven treatment option for many types of edentulism [ 1- 2]. Due to the promising properties of zirconia such as its aesthetic aspects, biocompatibility, strength, accuracy, and translucency, its use in implant-supported restorations is increasing [ 3- 5].

Two types of restorations may be used in implant cases; screw-retained or cement-retained [ 6]. Cemented restorations have some advantages such as better aesthetic aspects, passive fit, and lower fabrication costs. In addition, compared to screw-retained restoration, the fabrication process of cement-retained restoration is simpler and the creation of its occlusal morphology is more accurate. Meanwhile, cemented restorations exhibit more serious biological complications than screw-retained restorations (peri-implant inflammation as a consequence of possible remaining excess cement) and it is difficult to retrieve the restoration in the case of screw loosening, ceramic chipping, or evaluating the peri-implant tissue [ 7- 10]. The main advantage of screw-retained restorations is retrievability. In addition, they are more biocompatible because there is no excess cement in the sulcus. However, screw-retained restorations show more technical problems. The restoration screw might be fractured or loosened and the laboratory process for constructing them is more sophisticated and expensive. Due to the existence of screw access opening, creating an accurate occlusion is more difficult and the rate of porcelain fracture is higher [ 11- 15].

The main concern with cemented restorations is retrievability [ 16]. Several methods have been suggested to overcome this issue. Doerr [ 17] proposed the construction of a template to recognize the location of the screw. Daher *et al*. [ 18] used digital photographs to recognize the location of the screw. Another solution is using small lingual screws to attach the crown to the abutment [ 19]. Some investigators proposed placing a small stain on the layering ceramic at the location of the screw access opening [ 20]. All of the mentioned methods may be helpful in finding the location of the screw access hole. However, in all of this methods perforation of framework and veneering porcelain is necessary which may decrease the strength of the restoration [ 21]. Another suggestion is to use provisional cementation [ 22]. However, in using provisional cementation, the degree of retention is unpredictable and may require additional appointments for the patient to re-cement the restoration. 

In our previous study [ 23], we designed a ledge in the framework of cement-retained implant-supported metal ceramic restorations in the location of screw access hole to support remaining porcelain after perforation of framework. This design has the advantages of cement-retained restoration as well as the convenience of retrieval [ 23]. Due to increasing demands for all ceramic restorations in the current study, zirconia based implant supported crowns were tested. The purpose was to compare the strength of crowns which containing ledge with those without a ledge. There were two null hypotheses. First, preparing a hole in the location of abutment screw in zirconia based cement-retained restoration to provide has not any effect on the strength of it. Second, preparing a ledge in the site of the screw access hole on the coping of the zirconia-based cement-retained restoration would not prevent the weakening of the restoration due to the probable future perforation of the occlusal surface.

## Materials and Method

**Figure 1:**
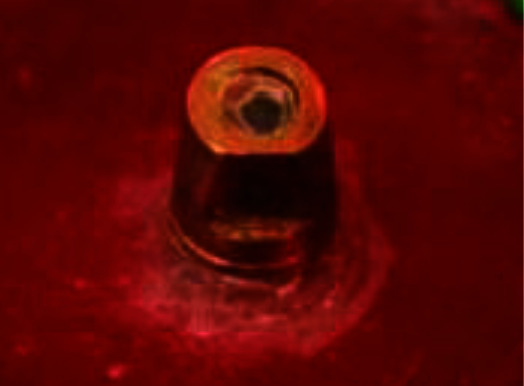
Brass die embedded in acrylic resin block

**Figure 2:**
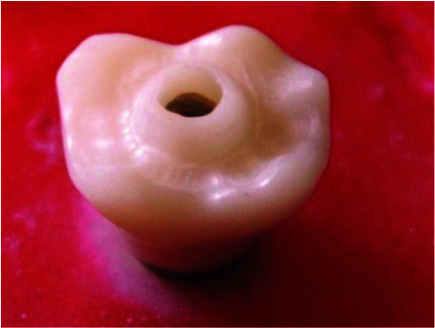
The ledge designed in the zirconia framework in the location of screw access channel of the third group

**Figure 3:**
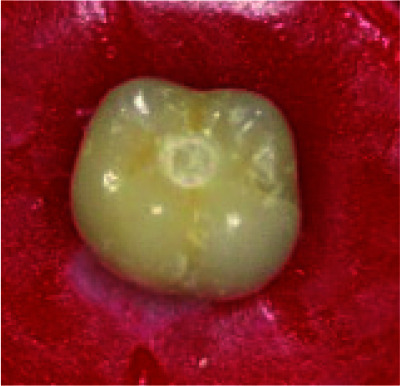
The holes in groups 2 and 3

An implant analog (DioCorp, Busan, South Korea) with the diameter of 5mm and the height of 6.5mm was connected to a straight titanium abutment (DioCorp, Busan, South Korea) with the diameter of 5mm, the height of 7mm, and the collar height of 3mm. This complex was used as a pattern to mill 33 brass dies exactly similar to model by using a lathe (CNC 350; Arix Co; Tainan Hesin, Taiwan). A total of 9.5mm of each sample was embedded in an acrylic resin block vertically (Figure 1). Subsequently, the dies were sprayed with scan spray and scanned using a 3D-laser scanner (3Shape D810; 3Shape, Copenhagen K, Denmark). The data were transferred to CAD software (3Shape's CAD Design software; 3Shape, Copenhagen K, Denmark). By considering a 30-µm space for the cement, a mandibular molar coping with a uniform thickness of 1mm around was designed. Twenty-two zirconia copings were milled from pre-sintered Y-TZP blanks (IPS Emax Zir CAD, Ivoclar Vivadent) in a milling machine (inLab MC, Sirona) and then sintered. Eleven samples were used for the control group and the other eleven samples were used for the second group (conventional). A 2mm diameter circle was drawn in the center of the occlusal table in the second group. Eleven zirconia frameworks were designed with the same sizes and shapes as those of the first and second groups except that a ledge was existed in the location of the screw access channel (Figure 2). The ledge was 1 mm in thickness and 1.5mm in height and it was located around a 2mm hole in the center of the occlusal surface. A silicone index was used to standardize porcelain application to all the samples. The crowns were then cemented by using zinc oxide-eugenol cement (TempBond; Kerr Mfg Co., Romulus, MI) to their corresponding dies. A 20-N load was applied during cementation for 15 minutes. A hole was prepared in the location of the abutment screw access channel in group 2 by using a 2 mm zirconia bur (Komet diamond bur, Lemgo, Germany) on a high-speed handpiece. Then the holes in groups 2 and 3 (designed in the framework) were filled with a photo-polymerized composite resin (3M ESPE Dental Products, Canada) (Figure 3). Afterward, all the crowns underwent thermal cycling for 500 cycles from 50°C to 65°C for 30 seconds with 12-second intervals to simulate oral conditions [ 24]. Finally, all the specimens were subjected to vertical static compressive load by using a universal testing machine (Zwick-Roell Z020; Zwick Gmb H &amp; Co. KG, Ulm, Germany) until they were fractured. The force was applied perpendicular to the occlusal surface in the central part of the crown by using the rounded edges of the loading piston a rate of 2 mm/min.

IBM SPSS statistical software (SPSS 22, IBM Corp) was used for data analysis. One-way ANOVA test was used for comparing the mean fracture resistance values of the samples. Then, pairwise comparison among the groups was done by using Tamhane post-hoc test. 

## Results

The least mean fracture resistance value was seen in group 2 (conventional) (960.09±210.67 N). In the special design group (group 3) this value was 1357.81± 361.68 N and in control group it was measured 1270.18±312.67 N (Table 1). The one-way ANOVA results showed statistically significant difference among the groups regarding the fracture resistance value (*p*= 0.011 and F=5.28). Tamhane post-hoc test was used to compare the fracture resistance values among the groups. The fracture resistance value was higher in the special design group than that of the conventional design (*p*= 0.018). The fracture resistance value of the conventional design group was less than that of the control group (*p*= 0.042). The value of mean fracture resistance between the control group and the special design group was not statistically different.

**Table1:** Measures of fracture resistance of crown specimens

Group	N	Mean	SD[Table-fn t1f1]*	Min	Max
Control	11	1270.18	312.67	919	1730
Conventional	11	960.09	210.67	672	1370
Special design	11	1357.81	361.68	913	2000

* Standard deviation

## Discussion

Regarding the results of the present study, the strength of the specially designed implant-supported cement-retained zirconia restoration was higher than that of the conventional design. Designing a supporting wall around the access hole in the framework of zirconia prevents the weakening of the restoration. In contrast, the restorations, which did not have this ledge and were perforated through the access hole showed a lower fracture resistance. Therefore, the null hypotheses were rejected. The presence of the screw access opening in screw-retained restoration has been shown to decrease the fracture resistance of it in several studies [ 21]. Karl *et al*. [ 25] concluded that the screw access opening of a metal-ceramic screw-retained restoration is a weak point in the ceramic layer.

In another study, Torrado *et al*. [ 26] found that a significantly lower force was needed to fracture screw-retained crowns than cement-retained crowns. Since in the current study a hole was created in the occlusal surface of the conventional group similar to the screw-retained crowns, the results of the present study may confirm those of the studies mentioned above. 

In some clinical situations, to retrieve the restoration, making a hole on the occlusal surface of the cement-retained implant-supported restoration is inevitable due to screw loosening or porcelain fracture. In the current research, a ledge was designed in the framework of the implant-supported zirconia-based cement-retained restoration. The goal of this special design was to prepare a support for the remaining veneering porcelain after creating a hole in the restoration. Analyzing the values in the present study showed that the crowns with the special design had better fracture resistance than those with the conventional design perforated by creating an access hole on their occlusal surface. Therefore, this feature may be used in case of high risk for screw loosening in patients with high force factors.

This study confirmed the results of a previous study done by Mokhtarpour *et al*. [ 27] they found that the fracture resistance of the implant-supported zirconia-based crowns which had a screw access hole was decreased. They claimed that the stage of preparation of the hole (before or after sintering) did not have any effect on the strength of the restoration. In one of the groups of the present research, the preparation of the hole was performed after sintering the samples. The fracture resistance values of these samples were less than that of the control group, which confirms Mokhtarpour *et al*.’s [ 27] study. In another group of the current study, a hole was created with a special design before sintering. The fracture resistance of these samples was similar to that of the control group. Thus, this result is inconsistent with that of Mokhtarpour *et al*. [ 27]. This difference may be related to the design of the hole. In the current study, a wall of zirconia, which could support the remaining porcelain after the creation of the hole, was designed in the framework.

The results of the current confirmed the results of a study performed by Sabury *et al*. [ 28]. They found that preparing a ledge in the position of the access hole did not have any effect on the strength of the restoration. However, in the current study, different brands of zirconia and veneering porcelain were examined. In contrast to the study of Sabury *et al*., in the current study, the preparation of the hole in the second group was done after sintering which was more similar to the clinical situations where the clinician is compelled to create a hole for the retrieval of the restoration after cementation. 

The special design of the hole proposed in the current study has the simultaneous advantages of cement restorations and retrievability. In addition to good strength, this design has some other advantages such as a more secure cementation due to better seating and excess cement removal. Because the height of the ledge was 1.5 mm in this study and the porcelain thickness on the occlusal surface of zirconia-based crowns is usually 1-1.5 mm, there was no worry about the interference of the ledge with occlusion. However, when the inter-arch space is limited, this design may not be used.

The design in the second group in this study was based on a common clinical situation when the clinician has to perforate the conventional restoration with a bur to retrieve the restoration. However, the fourth group could be added to the above-mentioned groups to evaluate the effect of microcracks which may be created during drilling with a high speed handpiece. 

 In the current study, a static compressive load was used to test the fracture resistance of the samples, whereas in a clinical setting, the crowns may undergo fracture following a dynamic load. Therefore, testing the samples under lateral, oblique, offset, and cyclic loads is suggested for future studies. In addition, only one type of temporary cement was used to cement the samples. Therefore, using different types of temporary and permanent cements is proposed.

In the current research, the ledge was formed in the framework of the molar tooth in the center of the occlusal surface. However, in clinical situations, the screw access hole may be in other points and other teeth. Thus, it is recommended that future studies focus on other teeth and creating a ledge in other points (for example, on a premolar functional cusp tip).

## Conclusion

Preparing an abutment screw access hole in the occlusal surface of cement-retained implant-supported zirconia-based crowns decreased the fracture resistance of the restoration. Designing a supportive ledge in the zirconia framework around the access hole may prevent the weakening of the restoration.
